# Prevalence and impact of pelvic floor dysfunctions on quality of life in women 5–10 years after their first vaginal or caesarian delivery

**DOI:** 10.1016/j.heliyon.2025.e42018

**Published:** 2025-01-21

**Authors:** Alba González-Timoneda, Nerea Valles-Murcia, Paula Muñoz Esteban, María Sol Torres López, Elisa Turrión Martínez, Patxi Errandonea Garcia, Lola Serrano Raya, Francisco Nohales Alfonso

**Affiliations:** a University and Polytechnic Hospital La Fe, Valencia, Spain; b University of Valencia (UV), Spain; c Nursing and Midwifery School of the Valencian Community, Spain; d Data Science Unit, Health Research Institute La Fe, Valencia, Spain; e Maternal Cardiovascular Health, Preeclampsia and Premature Birth, Health Research Institute La Fe, Valencia, Spain; f Neonatal Research Group, Health Research Institute La Fe, Valencia, Spain

**Keywords:** Pelvic floor disorders, Midwifery, Birth, Caesarean section, Pelvic floor

## Abstract

**Background:**

Pelvic floor dysfunction (PFD) in women is a health problem with repercussions on quality of life. The literature agrees that PFD prevention strategies begin with identifying women who are most at risk of developing PFD in the future. However, recent evidence addressing its multifactorial origin is scant.

**Objective:**

Our aim was to investigate late prevalence, risk factors, and the impact on quality of life of PFD in women after their first vaginal or caesarian birth.

**Methods:**

We conducted an ambispective cohort observational study. Participants were primiparous women who had given birth to only one child by vaginal delivery or cesarean section between 2012 and 2016. Exposure and response variables, assessed using the International Consultation on Incontinence Questionnaire and the Pelvic Floor Distress Inventory, were collected during a phone interview 5–10 years after childbirth. Pearson's Chi-square, Student's t-test and odds ratio (OR) with their respective 95 % confidence intervals (CI) were calculated.

**Findings:**

A total of 456 women were included in the study. Overall, 50 % of the women had 1 or more PFD within 10 years of giving birth, while 43.9 % of women presented urinary incontinence, 5.5 % presented pelvic organ prolapse, and 15.6 % of women reported some type of anal incontinence. A third of the women perceived the symptoms as a dysfunction and a half of them reported mild or moderate symptoms.

**Conclusion:**

PFD in women is a prevalent and underdiagnosed problem. Our study advocates for early detection of PFD risk factors and emphasizes the need for increased visibility, awareness, and proactive health measures related to PFD.

## Statement of significance

• Pelvic floor dysfunction in women is a health problem that affects millions of women globally, causing repercussions on their quality of life and generating considerable health costs.
• Numerous risk factors have been identified for the development of PFD, with vaginal delivery being the most important risk factor.
• There is a lack of recent data, and evidence is biased by investigating a population of women older than 60 years.
• Pelvic floor dysfunction is a prevalent health problem affecting almost 50 % of women within 10 years of giving birth.
• Women normalize pelvic floor dysfunction.

## 1 Introduction

Pelvic floor dysfunction (PFD), constitute a health problem that affects millions of women around the world and is associated with considerable financial costs for healthcare systems and women [1,2]. Urinary incontinence (UI), pelvic organ prolapse (POP), and anal incontinence (AI) are the PFD with the highest prevalence in the female population (25 % of women), with UI being the more frequent, with a prevalence of up to 17.1 % [3]. In previous studies, carried out years after birth, it is found a global prevalence of 35-38 % of PFD and a third of these women, having two or more PFD. Research on the relationship between childbirth and pelvic floor disorders is challenging because of the long latency for these disorders [4,5].

PFD adversely impacts various domains of women's life including psychological, physical, social and sexual wellbeing [6]. Numerous risk factors contribute to the development of PFD, including age, race, ethnicity, parity, education, poverty income ratio, body mass index, comorbidity count, and reproductive factors, vaginal birth being the most important risk factor for all these known disorders [7,8]. Although there are now many reports on PFD in women, only limited information is available when these disorders are concurrent [[9], [10], [11]].

Parity is considered one of the most important risk factors for PFD, and this is reflected in almost all major surveys. Thus, an epidemiological study using the 2005–2010 National Health and Nutrition Examination Survey with a total of 7924 non-pregnant women (aged 20 years and older) showed how increasing parity was also associated with symptoms of pelvic floor disorder. The risk increased from 1 to 1.6 in women with only 1 birth, increasing significantly after each new birth [12]. Perineal trauma, especially severe perineal tears, may also increase the risk of PFD where damage to these muscles can result in weakened support [13].

Interventions are available that may reduce risk of PFD development, such as weight reduction and pelvic floor muscle training (PFMT). However, researchers agree that PFD prevention strategies begin with identifying women who are most at risk of developing PFD in the future [14].

The existing knowledge about which obstetric risk factors should be assessed in relation to PFD is inconsistent. This is due to the multifactorial origin of PFD, in which the type of delivery and factors associated with delivery influence its pathogenesis. These associations may therefore be inconsistent if only isolated risk factors are taken into account. Research on the relationship between childbirth and PFD is challenging because of the long latency for these disorders.

The main aim of this study was to investigate late prevalence (at 5–10 years), risk factors, and the impact of PFD on quality of life in women after their first vaginal or caesarian delivery. In addition, grouping these PFD could also potentially be used to assess the long-term effects on quality of life and behaviors and attitudes of women.

## 2 Material and methods

### 2.1 Study design and participants

**Fig. 1:**
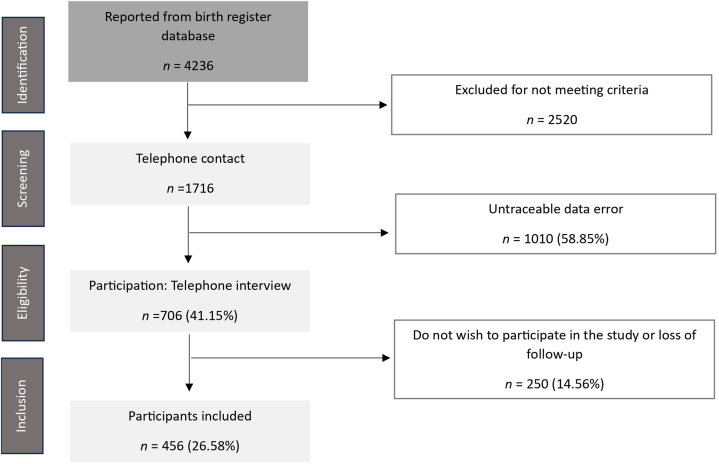
Flowchart depicting the selection process of women who gave birth to a single child between 2012 and 2016, as recorded in the La Fe University and Polytechnic Hospital database.

An ambispective cohort observational study was carried out. Participants were women who had given birth to only 1 child by vaginal delivery or caesarean section *(blinded*) between 2012 and 2016. Exclusion criteria were multiparity, multifetal, ongoing pregnancy, women with general illnesses, and women with a history of perinatal death. A description of the study population based on a flow chart and cohort characteristics, including an analysis of non-respondents, has been described in detail (Fig. 1).

### 2.2 Data collection

Exposure variables (sociodemographic, health, obstetric and pelvic floor variables) and response variables, assessed with the International Consultation on Incontinence Questionnaire Short Form (ICIQ-SF) [15] and the Pelvic Floor Distress Inventory Short Form (PFDI-20), Spanish version [16] were collected. Swimming, yoga, pilates and walking was considered low impact physical activity, whether running, crossfit or tennis were considered high impact sports.

The ICIQ-UI Short Form is a questionnaire for evaluating the frequency, severity, and impact on quality of life of urinary incontinence. The ICIQ-UI Short Form provides a score ranging from 0 to 21, with a higher score indicating greater severity of symptoms [14]. The PFDI-20 is comprised of 3 scales, which include the Urinary Distress Inventory-6 (UDI-6), Pelvic Organ Prolapse Distress Inventory-6 (POPDI-6), and the Colorectal-Anal Distress Inventory-8 (CRADI-8). The PFDI-20 includes 20 questions. Each question begins with a “yes” or “no” response. If “yes,” the patient must indicate how much bowl, bladder, or pelvic symptoms have been bothering them in the past 3 months on a 4-point scale that ranges from “not at all” (0) to “quite a bit” (4). The sum of the 3 scales is added together to get the PFDI-20 summary score, which ranges from 0 to 300 [16].

A complete database of births in primiparas, recorded between 2012-2016, was obtained from the hospital information system. First, medical records were reviewed to select participants according to the established selection criteria between November and December 2021. Women who had subsequent births, were currently pregnant or did not meet inclusion criteria any more were discarded. Secondly, eligible women were called by telephone to reconfirm that they still met criteria and to invite them to participate in the study (Fig. 1) Email addresses from women who agreed to participate were collected, and information related to the study, the informed consent and the questionnaires was sent. In addition, exposure variables data were collected in a brief telephone interview. Data collection was carried out between January and September 2022.

### 2.3 Quantitative analysis

Statistical analysis was performed using *Stata Statistical Software (Release 13. College Station, TX: Stata Corp LP).* Prevalence and 95 % confidence intervals (CI) were calculated. Chi-squared test was used to compare categorical variables and Student's test to compare continuous variables. Multivariate analysis with logistic regression was conducted to analyze independent risk factors for isolated and combined PFD, depending on the age at delivery, current age, body mass index (BMI) at delivery and current, educational level, physical exercise, work occupation, type of delivery, newborn weight, and performance of an episiotomy. The *odds ratio* (OR) and its 95 % CI were calculated from the logistic regression model. A *p*-value of <0.05 was considered statistically significant.

## 3 Results

A total of 456 women (64.6 %) agreed to participate in the study (Fig. 1). The average age of the participating women at the time of delivery was 33.1 years. Sociodemographic and obstetric characteristics of the sample are shown in Table 1.

**Table 1:** Sociodemographic and obstetric characteristics of the participants.

	Mean (min/max)	N	%
**Age at birth**	33.1 (16–51)		
**Current age**	41.6 (26–60)		
**Education level**			
*High (secondary/university)*		366	80.2
*Basic*		10	2.1
**Job occupation**			
*Weight load*		213	46.7
**Family history**			
*Mother history*		91	19.9
*Sister history*		16	3.5
**Physical exercise**		329	72.2
***With impact***		89	19.5
*2–3/week*		202	61.4
**Constipation**		80	17.5
**Current pathology**		66	14.5
**Menopause** **Body Mass Index (BMI)**		31	6.8
*Current BMI*	24.1 (15.6–43.5)		
*Pregestational BMI*	22.9 (15.2–42.8)		
*Birth BMI*	27.5 (16.9–43.8)		
**Weight gain**	12.2 (-12–40)		
**Gestational age**	39 (26–42)		
**Newborn weight**	3168 (850–4900)		
**Type of delivery**			
***Eutocic***		214	46.9
***Instrumental***		132	28.9
*Obstetric Suction Cup*		101	
*Forceps*		17	
*Spatulas*		14	
**C-section**		110	24.1
Episiotomy		289	83.5

A quarter of women, *n* = 110 (24.1 %), had undergone caesarean section and the rest of womenn *n* = 346 (75.8 %) had given birth vaginally; *n* = 214 (61.8 %) of deliveries were normal and *n* = 132 (38.2 %) instrumental. Among the instrumental deliveries, *n* = 101 (76.5 %) had been performed with an obstetric vacuum, *n* = 17 (12.9 %) with forceps, and *n* = 14 (10.6 %) with spatulas. An episiotomy was performed in *n* = 289 (83.5 %) of vaginal deliveries, and no records of type III and IV tears were found. However, up to 7 women (2 % of vaginal deliveries) suffered fecal incontinence without a diagnosis of causal medical disease.

Regarding the prevalence of PFD, on the one hand, when women were asked about the presence of urinary incontinence, fecal incontinence, pelvic organ prolapse, or pelvic pain/dyspareunia, more than two thirds of women *n* = 308 (67.5 %) reported not having any type of PFD, compared to *n* = 135 (29.6 %) who reported having one type of PFD and *n* = 13 (2.8 %) who reported having 2 or more types of PFD. Of those women who reported having some type of PFD, *n* = 10 (6.7 %) reported its presence before pregnancy, *n* = 5 (3.4 %) reported its appearance during pregnancy, and *n* = 120 (89.9 %) after pregnancy. The mean time of appearance of PFD was 2.8 years after pregnancy.

**Fig. 2:**
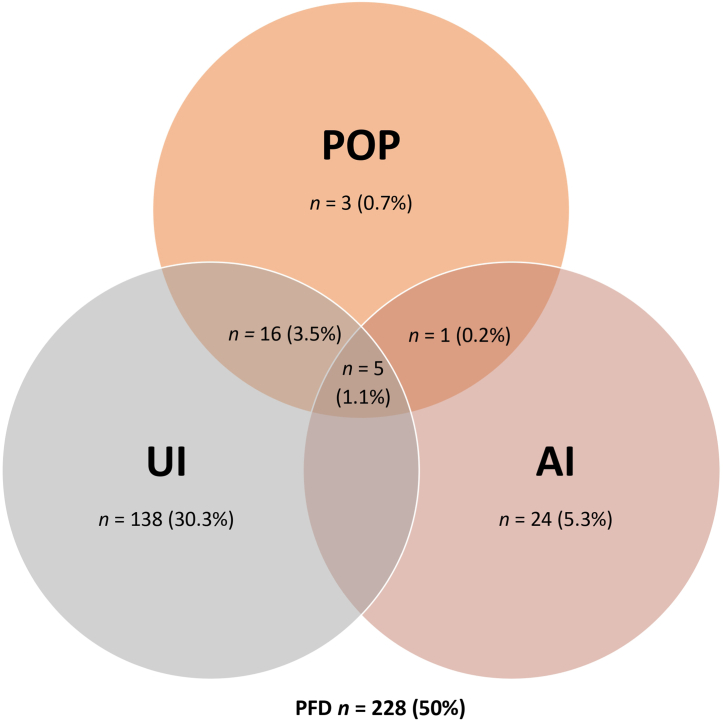
Venn diagram showing the prevalence of urinary incontinence (UI), pelvic organ prolapse (POP), and anal incontinence (AI), as well as their overlap, illustrating the total combined prevalence.

In terms of the results obtained from the validated tests, 50 % (*n* = 228) of the total cohort reported any PFD within 10 years of giving birth. Fig. 2 shows a Venn diagram of the overlapping prevalence of urinary incontinence, pelvic organ prolapse, and anal incontinence in primiparous women 5–10 years after 1 vaginal delivery or caesarian section.

According to the validated ICQ_SF questionnaire, *n* = 197 (43.2 %) had some type of urine loss in the last month, occurring once a week in *n* = 118 (25.8 %) of women, at least 3 times a week in *n* = 47 (10.3 %) of women, and at least once a day in *n* = 8 (1.7 %) of women. In terms of the UDI-6A, the prevalence of urinary incontinence was 43.9 % (*n* = 200). Of these women, *n* = 96 (21.1 %) reported involuntary urine loss associated with a feeling of urgency, while *n* = 171 (37.5 %) reported involuntary urine leaks when coughing, sneezing, or laughing. In either case, urine incontinence was not considered an important discomfort by *n* = 50 (51.8 %) and *n* = 91 (53.2 %) of those women, respectively.

In response to the POPDI-6, *n* = 25 (5.5 %) of the participants reported “feeling a bulge” outside the vagina, which was perceived as asymptomatic in *n* = 15 (60 %) of the women. A total of *n* = 71 women (15.6 %) reported some type of anal incontinence (gas, liquid, or solid stools). Five (*n* = 5) women (1.1 %) reported involuntary fecal losses of solid stools, *n* = 11 (2.4 %) reported involuntary fecal losses of soft or liquid stools and *n* = 65 women (14.3 %) mentioned involuntary gas losses. Less than half of the women who had involuntary fecal loss of soft stools *n* = 5 (45.4 %) or gas losses *n* = 31 (47.7 %) were not bothered, whereas all women perceived involuntary fecal losses of solid stools as very annoying.

Our study revealed no association between current age and age at the time of delivery and the presence of pelvic organ prolapse symptoms. Similarly, current BMI does not correlate with the presence of symptoms consistent with pelvic organ prolapse. No significant differences were observed with regard to level of education. In terms of type of birth, the group of women who had a caesarean delivery were seen to present statistically fewer pelvic organ prolapse symptoms [95 % CI, OR 0.11 (0.14–0.86) (*p* = 0.03)]. Nevertheless, the weight of the newborn, the practice of physical exercise and the performance of an episiotomy do not have a statistically significant influence on the appearance of symptoms consistent with pelvic organ prolapse. The correlation of pelvic organ prolapse symptoms after first birth with obstetric and demographic variables is shown in Table 2.

**Table 2:** Correlation of pelvic organ prolapse symptoms after first birth with obstetric and demographic variables. Multivariate analysis with adjusted Odds Ratios (aOR) and 95 % Confidence Intervals (CI).

	N (25)^a^	%	aOR (IC95 %)	p
Age at delivery				
<30	6	24	1	
30-35	8	32	0.68 (0.23–2.03)	0.49
>35	11	44	1.33 (0.47–3.73)	0.57
Education level				
Low (none/basic)	4	16	1	
High (secondary/university)	21	84	1.30 (0.43–3.91)	0.63
Physical exercise				
None	3	12	1	
Low impact	17	68	3.15 (0.90–10.96)	0.07
High impact	5	20	2.46 (0.57–10.57)	0.22
Current BMI				
<18.5	1	4	1	
18.5–29.9	21	84	0.99 (0.12–7.83)	0.99
>30	3	12	1.5 (0.15–16.39)	0.69
Type of delivery				
Eutocic	16	64	1	
Instrumental	8	32	0.79 (0.33–1.92)	0.61
C-Section	1	4	0.11 (0.14–0.86)	**0.03∗**
Newborn weight				
<2800g	2	8	1	
2800–3500g	17	68	3.12 (0.70–13.82)	0.13
>3500g	6	24	1.95 (0.38–9.90)	0.41
Episiotomy				
No	5	20.8	1	
Yes	19	79.2	0.74 (0.26–2.08)	0.57

a Women who had a cesarean delivery exhibited statistically (*p* < 0.03) fewer symptoms of pelvic organ prolapse (POP) compared to those who had a vaginal delivery, based on the analysis of the data.

Age at the time of delivery was significantly higher (*p* = 0.03) in the group of women who had AI. However, when this analysis is carried out by age groups, no statistical significance is observed. Instead, from a statistical perspective, the current age of respondents at the time of the interview was higher in all types of anal incontinence (*p* = 0.01). Jobs involving heavy loads represent a statistically significant protective factor for the appearance of anal incontinence [95 % CI, OR 0.52 (0.30–0.89) (*p* = 0.02)]. No statistical significance was found to correlate level of education, BMI at the time of the interview, route of birth, weight of the newborn, performance of episiotomy and practice of physical exercise with the appearance of anal incontinence symptoms. The association of the selected variables with anal incontinence is shown in Table 3.

**Table 3:** Correlation of anal incontinence symptoms after the first birth with obstetric and demographic variables. Multivariate analysis with adjusted Odds Ratios (aOR) and 95 % Confidence Intervals (CI).

	N (71)	%	aOR (IC95 %)	*p*
Age at delivery				
<30	11	15.5	1	
30-35	33	46.5	1.68 (0.81–3.48)	0.15
>35	27	38	1.92 (0.90–4.08)	0.08
Education level				
Low (none/basic)	16	22.5	1	
High (secondary/university)	55	77.5	0.81 (0.44–1.50)	0.52
Occupation				
Unloaded work	47	66.2	1	
Weight load	24	33.8	0.52 (0.30–0.89)	**0.018**^a^
Physical exercise				
None	15	21.1	1	
Low impact	44	62	0.67 (0.89–3.14)	0.10
High impact	12	16.9	1.16 (0.51–2.62)	0.71
Current BMI				
<18.5	2	2.8	1	
18.5–29.9	62	87.7	1.5 (0.35–6.91)	0.55
>30	7	9.9	1.9 (0.36–10.64)	0.42
Type of delivery				
Eutocic	32	45.1	1	
Instrumental	18	25.3	0.89 (0.48–1.67)	0.73
C-Section	21	29.6	1.34 (0.73–2.46)	0.34
Newborn weight				
<2800g	15	21.1	1	
2800–3500g	33	46.5	0.73 (0.37–1.43)	0.36
>3500g	23	32.4	0.97 (0.47–1.98)	0.93
Episiotomy				
No	9	18	1	
Yes	41	82	0.75 (0.45–1.26)	0.28

a Jobs involving heavy loads appear to serve as a statistically significant protective factor against the development of anal incontinence.

Age at the time of delivery appears as a risk factor for the appearance of total urinary incontinence (*p* ≤ 0.001). When statistical analysis is performed by age groups, we find that there is statistical significance for the group older than 35 years compared to the group younger than 30 years [95 % CI, OR 2.05 (1.22–3.43) (*p* ≤ 0.001)]. If stress urinary incontinence (SUI) and urgency urinary incontinence (UUI) are analyzed separately, we see this significance in SUI compared to UUI, *p* ≤ 0.001 and *p* = 0.05 respectively in both groups.

Thus, it has been observed that as age increases, there is a significant and probabilistic correlation with the appearance of incontinence associated with sphincter support components. A significant difference has been found in the current age at the time of the interview for total urinary incontinence (*p* = 0.001), as well as between the group of women with SUI and the group that does not present incontinence (*p* ≤ 0.001). However, this difference is not observed in the case of UUI.

A high level of education, defined as completion of high school, vocational training, or university studies, acts as a protective factor against the appearance of urinary incontinence [95 % CI, OR 0.55 (0.34–0.88) (*p* = 0.01)]. If we analyze both incontinences separately, we find statistical significance for SUI (*p* = 0.03). However, this protective association has not been found in the case of UUI.

No significant differences were found in current BMI, physical exercise, type of delivery and episiotomy between the groups of women with incontinence and those who did not present symptoms, since the values were very similar in both groups. The association of the selected variables with anal incontinence is shown in Table 4.

**Table 4:** Correlation of urinary incontinence symptoms after the first birth with obstetric and demographic variables. Multivariate analysis with adjusted Odds Ratios (aOR) and 95 % Confidence Intervals (CI).

	N (200)	%	aOR (IC95 %)	*p*
Age at delivery				
<30	36	18 %	1	
30-35	88	44 %	1.5 (0.92–2.44)	0.103
>35	76	38 %	2.05 (1.22–3.43)	**0.006**^a^
Education level				
Low (none/basic)	50	25	1	
High (secondary/university)	150	75	0.55 (0.34–0.88)	**0.013∗∗**
Physical exercise				
None	62	31	1	
Low impact	106	53	0.82 (0.53–1.27)	0.39
High impact	32	16	0.58 (0.33–1.02)	0.06
Current BMI				
<18.5	10	5	1	
18.5–29.9	168	84	0.6 (0.25–1.63)	0.36
>30	22	11	1.32 (0.43–4.02)	0.62
Type of delivery				
Eutocic	97	48.5	1	
Instrumental	65	32.5	1.1 (0.75–1.80)	0.4
C-Section	38	19	0.63 (0.39–1.02)	0.06
Episiotomy				
No	21	13	1	
Yes	141	87	1.67 (0.93–3)	0.08

∗UI = PFDI-20_16 or PFDI-20_17.

∗Maternal age at the time of delivery is a statistically significant risk factor (*p* ≤ 0.006) for the onset of total urinary incontinence, including both stress urinary incontinence (SUI) and urge urinary incontinence (UUI), in women aged >35 years.

∗∗ A higher level of education, defined as the completion of secondary education, vocational training, or higher education, acts as a statistically significant protective factor (*p* < 0.013) against the onset of stress urinary incontinence.

## 4 Discussion

Substantial progress has been made in reducing maternal mortality worldwide over the past three decades. However, the historical focus on mortality reduction has been accompanied by a comparative neglect of complications of labor and birth that may arise or persist months or years after birth. However, if we review the epidemiological data on medium and long-term complications arising from labor and birth beyond 6 weeks on maternal health, we find that conditions affecting more than 10 % of women in the postpartum period in the medium or long term include dyspareunia; anal or urinary incontinence, or both; postpartum depression; tokophobia (intense fear of childbirth); and chronic postpartum pain, such as low back pain and perineal pain. On the other hand, conditions affecting between 1 % and less than 10 % of women include pelvic organ prolapse, postpartum post-traumatic stress disorder (PTSD) related to childbirth, postpartum thyroid dysfunction, lactational mastitis, HIV seroconversion, and nerve injury to the lower extremities [17]. Our study has been limited to pelvic organ prolapse, urinary and anal incontinence.

Overall, half of the women included in our study reported 1 or more PFD within 10 years of giving birth. These figures are similar to the ones shown in the SWEPOP study [18], as also shown in other studies [11], while differ from prevalence studies in United States [12]. Evidently, these studies compare populations with different characteristics and the instruments used for measuring prevalence are also different. However, all studies do agree that urinary incontinence is the most prevalent dysfunction.

In our study, dual dysfunction was found in 12.7 % of women. This combination was a significant predictor of an increased risk of concurrent incontinence, such as urinary and anal (gas) incontinence. Altman [19] found that most women with urinary incontinence also experienced flatus incontinence, regardless of delivery mode. In a Swedish cohort study [20], a questionnaire on medical history, urinary and fecal incontinence, and genital prolapse symptoms was mailed to 1000 women in their 40s and 1000 women in their 60s. Univariate analyses revealed statistically significant associations between urinary incontinence and gas incontinence [4.8 (3.0–7.8)], liquid stool incontinence [5.0 (2.9–8.6)], and solid stool incontinence [5.9 (2.4–14.2)]. In our study, the combination of the three types of DSP occurred in only 1 % of cases, compared to 2.9 % in the SWEPOP study [18].

The average age of women (33.1 years) at the birth of their first child in our setting corresponds to the official state records [21]. However, the proportion of caesarean sections in our setting is higher due to the characteristics of the pregnant women attending our hospital, which is a third level hospital that addresses higher complexity.

Our study revealed a prevalence of 43.9 % of women who responded affirmatively to having had episodes of urinary incontinence in the last month. These results are similar to those of another Spanish study [22] that reported prevalences of UUI and SUI of 21.4 % and 39.6 %, respectively. Our results are also in line with other cohorts found in the literature with UUI and SUI prevalences of 19.2 % and 43.6 % respectively 5 years after the first delivery [23] or an SUI prevalence of 42 %, 12 years after the first delivery [24].

Age at the time of delivery, as well as age at the time of the interviews, emerged as risk factors for the appearance of total urinary incontinence and SUI, but were not risk factors for UUI. A statistical analysis performed by age group showed statistical significance for the group older than 35 years compared to the group younger than 30 years. In other studies, age also influences the occurrence of urinary incontinence, but the inclusion of older populations, including mainly menopausal women, introduces new confounding factors [11]. A high-level education, defined as completion of secondary education, vocational training, or university studies, acted as a protective factor against the occurrence of global urinary incontinence or SUI. In contrast, a meta-analysis by Hage-Fransen [25] found that a low level of schooling did not reach statistical significance [OR 1.16 (0.52–2.59)] as a risk factor for urinary incontinence. However, few studies have included this variable.

What is expected from the literature is a relationship between BMI and clinical urinary incontinence. However, due to the characteristics of our sample, with a mean age of 41.6 years and a prevalence of obesity (BMI > 30) of 11 %, our study is not comparable with other studies whose mean population is 20 years older [26,27]. Regarding the route of delivery and the occurrence of any type of incontinence, our study shows a higher risk associated with instrumental delivery and a lower risk associated with cesarean delivery compared to normal vaginal delivery. In line with our study, Gyhagen [4], also confirmed that caesarean delivery would have a protective effect.

It was observed that age at delivery was significantly higher in the group of women who presented anal incontinence, which was maintained at the time of the interview. We found no other differences with respect to the variables studied. Furthermore, in our cohort study we did not find caesarean section to be a protective factor in the occurrence of anal incontinence, similar to the results of the meta-analysis by Nelson [28]. The statistical relationship between anal incontinence and anal sphincter injury is of interest. A Norwegian prospective population-based cohort study [29] showed that obstetric anal sphincter injury increased the risk of fecal incontinence and gas [OR 4.1 (1.7–9.6)] after delivery. This statement was confirmed in the Hage-Fransen meta-analysis [25] with related factors up to 18 months postpartum. However, this relationship is not well documented over the years (between 4 and 12 years), so this conclusion should be evaluated with caution. Studies show significant ORs from pooled univariate data with >10,000 participants and at least 5 studies of forceps/vacuum extraction, maternal age >35 years, and spontaneous vaginal delivery. Along the same lines, in a 12-year longitudinal study, women who had one or more forceps deliveries were more likely to have persistent anal incontinence [OR 2.08 (1.53–2.85)], while more obese women than normal weight women reported persistent incontinence [OR 1.52 (1.06–2.17)] [30,31].

In our series we have not described any grade III or IV perineal tears. However, we identified up to 7 women (2 %) who presented fecal incontinence after vaginal delivery and did not receive subsequent follow-up. This percentage coincides with the obstetric anal sphincter injuries (OASIS) rate in our maternity ward (internal data file of the Pelvic Floor Unit of *(blinded)*. This highlights the possibility of underdiagnosis of tears of this type.

In our study, 25 women (5.5 %) reported symptoms compatible with pelvic organ prolapse. The mean age of these women was 41.6 years, with only 6.8 % being in menopause, which offers a good estimate of the prevalence of this dysfunction while avoiding the effects of aging. In this respect, a Dutch cross-sectional population-based study [2], estimated the prevalence of pelvic organ prolapse symptoms in women with a mean age of 58 years, and found that 72.9 % of women were menopausal. These women were much older than the participants in our study. The study [2] reported a prevalence of vaginal bulging sensation in 12.2 %, regardless of the symptoms experienced. Subsequently, after physical examination of the participants, no exact correlation was obtained between symptomatology and the degree of existing prolapse (17.5 % had POPQ > 2B), a discordance that has been studied in depth in the literature [[32], [33], [34]].

Of the different variables analyzed, only women who had a caesarean delivery presented statistically fewer pelvic organ prolapse symptoms [95 % CI, OR 0.11 (0.14–0.86) (*p* = 0.03)]. These data correspond with previous studies, where the protective effect of caesarean section on pelvic organ prolapse is analyzed. Thus, the prevalence of pelvic organ prolapse doubled after vaginal delivery compared to caesarean section 2 decades after a delivery; newborn birth weight and respondents’ current BMI were risk factors for pelvic organ prolapse after vaginal delivery [4]. However, caesarean section would not give universal protection, due to a possible genetic predisposition that may also partly explain the occurrence of PFD in nulliparous women and those who have undergone caesarean delivery.

Studying the impact of PFD on quality of life the severity of urinary incontinence, understood as daily SUI, was reported by 7.7 % of women, coinciding with Altman [19]. For Viktrup [24], daily SUI was reported at a lower rate of 5.4 %, while in a multicenter study conducted in 3 maternity wards [30], the rate of daily or more frequent SUI at 12 years postpartum was 14.3 %. Using the PFDI scale, 24.6 % of these cases were found to be very bothered by urine leakage, but only 19 women (4.2 %) had received physiotherapy treatment for PFD, and only 2 women required tension-free vaginal tape anti-incontinence surgery.

Another noteworthy fact is that in cases of anal incontinence, most women were severely affected. This percentage is similar to the 69.2 % of women who were affected in cases of gas incontinence. In cases of pelvic organ prolapse (*n* = 25), only 1 patient required surgery.

When asked directly via telephone interview, one third of women answered affirmatively that they had PFD of any type. However, when the questionnaires were evaluated, 50 % of the interviewees reported being affected by PFD (single or combined). This fact could support the hypothesis that there is normalization of PFD and, consequently, underdiagnosis. Although it is known that women with PFD experience a series of problems that impact different spheres of their quality of life, not all women have these problems or do not express it for fear of what others may say, as reported in previous studies [[35], [36], [37]]. The Royal College of Obstetricians and Gynecologists [38] showed there remain significant barriers to seeking help, including a lack of knowledge about pelvic floor health and embarrassment surrounding the symptoms of PFD.

In at least half of the cases, the impact of PFD on quality of life is generally low or zero, leading to a lack of motivation for consultation and subsequent treatment. Unfortunately, though, there is a normalization of symptoms in young women, a fact that is also reflected in recent literature [39]. We must therefore focus on those known pelvic floor risk factors, and although our environment (age and route of delivery) offers little room for improvement, we believe that we must implement secondary prevention strategies before new events (subsequent pregnancies and births or aging) increase the prevalence of PFD. In this regard, the Cochrane review provides evidence that PFMT is more beneficial than placebo, no treatment or usual care for all types of urinary incontinence with respect to healing or improvement of outcomes and quality of life. PFMT is an effective treatment for urinary incontinence [40] that significantly improves the quality of life of women with this problem [41,42]. There is also moderate evidence that if PFMT is more intense, more frequent, delivered with individual supervision, its effectiveness improves, irrespective of combination with behavioral interventions [40]. As PFMT have no known side effects and many women present both pelvic organ prolapse and SUI, PFMT should also be recommended in combination with other treatments [43,44]. Midwives should continue to encourage regular and frequent practice of pelvic floor exercises in the postnatal period and beyond. It is essential to investigate and recognize the impact of perineal pain, stress incontinence or other PFD on women's daily life and psychological and emotional wellbeing at the postpartum checkup.

Finally, the main strength of our study is the long follow-up period, with a high response rate of women between 5 and 10 years after childbirth (64.6 %). Furthermore, our study only included primiparous women without subsequent births, which is the best model to investigate the long-term effects of pregnancy and childbirth in terms of PFD.

In order to minimize selection bias, selection criteria were clearly defined from the beginning of the study. Furthermore, observer bias and recall bias were minimized by using standardized questionnaires and collecting exposure data from medical records. Each short-form scale used (Urinary Distress Inventory, Pelvic Organ Prolapse Distress Inventory and Colorectal-Anal Distress Inventory) demonstrates a significant correlation with the long-form scale (*r* = 0.86, *r* = 0.92 and *r* = 0.93, respectively, *p* ≤ 0.001). These are a short, valid, and reliable form of specific questionnaires that measure the quality of life of women with pelvic floor disorders.

However, the findings of this study have to be seen in light of some limitations. The fact that some data were not obtained in personal interviews but through online questionnaires sent by email, as well as the absence of physical examination can be considered a limitation of the study.

## 5 Conclusions

This study is the first large-scale Spanish research on PFD at least five years postpartum, with 456 valid interviews. Our research has conclusively shown that PFD is a prevalent health problem affecting almost half of women after childbirth. Older age at delivery and at the time of interview, higher current BMI, instrumental delivery and episiotomy are risk factors associated with urinary incontinence. Higher educational level was found to be a protective factor for urinary incontinence. Women with caesarean section have statistically fewer pelvic organ prolapse symptoms, and the practice of physical exercise, even if of low impact, correlates with the presence of POP symptoms.

Based on perceived health, the findings indicate that DSP symptoms are normalized within the study population. Only a third of the cohort perceived themselves to have PFD, although the assessment tests concluded that this figure rises to half of the cohort. This fact is evidenced by the low percentage of women who request specialized health care.

In conclusion, PFD in women is a prevalent and underdiagnosed problem. Our study advocates for early detection of PFD risk factors and emphasizes the need for increased visibility, awareness, and proactive health measures related to PFD.

## CRediT authorship contribution statement

**Alba González-Timoneda:** Writing – review & editing, Writing – original draft, Supervision, Project administration, Methodology, Funding acquisition, Formal analysis, Data curation, Conceptualization. **Nerea Valles-Murcia:** Writing – review & editing, Writing – original draft, Project administration, Data curation. **Paula Muñoz Esteban:** Writing – original draft, Data curation. **María Sol Torres López:** Writing – original draft, Data curation. **Elisa Turrión Martínez:** Writing – original draft, Data curation. **Patxi Errandonea Garcia:** Writing – original draft, Data curation. **Lola Serrano Raya:** Data curation, Conceptualization. **Francisco Nohales Alfonso:** Writing – review & editing, Supervision, Project administration, Methodology, Funding acquisition, Formal analysis, Conceptualization.

## Ethics and consent declarations

This study was reviewed and approved by the Drug Research Ethics Committee - the approval number: 2021-855-1, dated November 15, 2021. All participants provided written informed consent to participate in the study and for their data to be published. Informed consent was obtained from each participant objectively, voluntarily, and with the possibility of revocation at any time, after they had received exhaustive information regarding the project by telephone and via email.

## Funding

This study was funded by a research grant awarded by the Official College of Nursing of Valencia, Spain.

## Declaration of competing interest

The authors declare that they have no known competing financial interests or personal relationships that could have appeared to influence the work reported in this paper.
